# Early Lymph Node Metastasis May Predict Poor Prognosis in Soft Tissue Sarcoma

**DOI:** 10.1155/2019/6708474

**Published:** 2019-12-12

**Authors:** Makoto Emori, Hiroyuki Tsuchie, Hiroyuki Nagasawa, Tomoko Sonoda, Arihiko Tsukamoto, Junya Shimizu, Yasutaka Murahashi, Emi Mizushima, Kohichi Takada, Kazuyuki Murase, Kotoe Iesato, Keita Igarashi, Tsukasa Hori, Masaki Yamamoto, Shintaro Sugita, Naohisa Miyakoshi, Tadashi Hasegawa, Yoichi Shimada, Toshihiko Yamashita

**Affiliations:** ^1^Department of Orthopedic Surgery, Sapporo Medical University School of Medicine, Sapporo, Hokkaido 060-8543, Japan; ^2^Department of Orthopedic Surgery, Akita University School of Medicine, Akita, Akita 010-8543, Japan; ^3^Department of Public Health, Sapporo Medical University School of Medicine, Sapporo, Hokkaido 060-8543, Japan; ^4^Department of Medical Oncology and Hematology, Sapporo Medical University School of Medicine, Sapporo, Hokkaido 060-8543, Japan; ^5^Department of Pediatrics, Sapporo Medical University School of Medicine, Sapporo, Hokkaido 060-8543, Japan; ^6^Department of Diagnostic Pathology, Sapporo Medical University School of Medicine, Sapporo, Hokkaido 060-8543, Japan

## Abstract

**Background:**

Lymph node metastasis (LNM) is a relatively rare event in soft tissue sarcoma. An association between the timing of LNM detection and patient prognosis is presently unknown.

**Patients and Methods:**

We retrospectively analyzed the clinicopathological features of 33 patients with LNM between 2001 and 2015. Analysis of the timing of LNM diagnosis was grouped according to patients presenting LNM in either <8 months (the median time from primary tumor diagnosis to LNM) or ≥8 months after primary tumor diagnosis.

**Results:**

A relationship between the primary tumor size and the timing of the LNM was not significantly found (*Rs* = 0.0088, *p*=0.96). Sixteen patients had an LNM detection duration of <8 months, and 17 patients had a duration of ≥8 months. The 5-year survival for patients with an LNM detection duration of <8 months and ≥8 months was 19% and 71%, respectively (*p*=0.0016). There were 19 patients with pulmonary metastases. Among them, there were 13 patients with a duration of primary tumor diagnosis to LNM of <8 months and 6 with a duration of ≥8 months (*p*=0.01).

**Conclusion:**

Early LNM (<8 months) may predict poor prognosis in soft tissue sarcoma.

## 1. Introduction

Distant metastasis is common in patients with soft tissue sarcoma (STS) of the extremities, occurring in about 25% of patients [1, 2]. Pulmonary metastasis is the most common form of metastatic disease. The median survival after a diagnosis of distant metastatic disease was approximately 12 months, with a 5-year survival of about 10% [3]. However, several studies have suggested that surgical management of pulmonary metastasis has achieved 5-year survival rates of 30% or more [4, 5].

Lymph node metastasis (LNM) is a relatively rare event in STS, except for lymphogenous histotypes such as rhabdomyosarcoma, epithelioid sarcoma, and clear cell sarcoma [6]. The overall prevalence is 1.75–12% with an increasing frequency due to the implementation of FDG-PET for whole body screening [7, 8]. Regional LNM is an important prognostic factor in STS, suggesting that LNM represents a component of disseminated disease [7, 9]. Unlike pulmonary metastasis, the precise role of lymphadenectomy remains to be defined because LNM in patients with STS is rare, thereby limiting the feasibility of well-designed prospective studies to establish the role of lymphadenectomy. There are some reports describing an association between the time from the primary STS diagnosis to LNM and patient prognosis [10, 11]. However, the precise duration, from primary diagnosis to detection of LNM, affecting overall survival (OS) is unknown.

The objective of this study was to analyze the clinicopathological features and patterns of LNM, focusing on the time duration from the primary diagnosis to the detection of LNM.

## 2. Patients and Methods

### 2.1. Patients

We retrospectively reviewed the oncology/reconstruction surgery database of our institutions. Thirty-three patients with LNMs who were treated at two hospitals (Sapporo Medical University Hospital and Akita University Hospital) between 2001 and 2015 (19 males and 14 females, with a median age of 61 years) were enrolled. The time to metastasis was defined as the period from the initial diagnosis of the primary tumor to the detection of LNM. The surgical stage was classified according to the seventh edition of the tumor-node-metastasis (TNM) classification of the International Union Against Cancer at initial presentation. The histological grade was determined on the basis of the French Federation of Cancer Centers (FNCLCC) grading system. This classification is based on the mitotic index, extent of necrosis, and degree of histological differentiation of the tumor. This study was approved by the Institutional Review Board for Clinical Research at our universities. Patients were reviewed at 3-month intervals until 3 years after the primary tumor diagnosis, then at 6-month intervals until 5 years after the primary tumor diagnosis, and then at 12-month intervals for the rest of the patient's life. A computed tomography scan of the chest and a localized magnetic resonance imaging scan were performed every 3 months during the third year after primary tumor diagnosis and every 6 months thereafter.

### 2.2. Definition and Treatment Strategy of LNM

We defined LNM as growing nodes and irregular borders, as detected on CT or MRI (*n* = 6), or diagnosed as LNM after biopsy or resection (*n* = 27). Patients with isolated LNMs were mainly treated with surgery as the primary modality. Patients with a surgically inaccessible location, extensive nodal disease, or systemic dissemination were treated primarily with chemotherapy or radiotherapy.

## 3. Statistical Methods

OS was defined as the time interval between the date of initial patient presentation and either the date of death from any cause or the date of the last patient contact. Spearman rank correlation coefficients were used to determine any association between the primary tumor size and the timing of LNM diagnosis. Fisher's exact test and Mann–Whitney *U* test were used to compare the timing of LNM diagnosis and the clinicopathological factors. Logistic regression was performed for analyzing the relationship between the pulmonary metastases as a dependent variable and the timing of LNM diagnosis as an independent variable. OS was estimated using the Kaplan–Meier method, with any differences in survival being determined using the log-rank test. A probability of *p* < 0.05 was considered statistically significant. Statistical analyses were performed with SPSS, version 23 (IBM Corp., Armonk, NY, USA).

## 4. Results

### 4.1. Clinicopathological Characteristics

**Figure 1:**
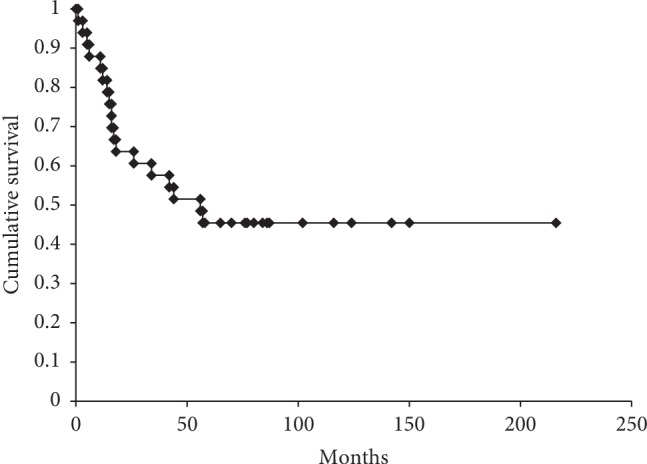
5-year OS rate of patients with lymph node metastases.

Patient demographics, primary diagnosis, tumor site, tumor size, tumor grade, surgical stage, treatment for the primary tumor, type of surgery, and pulmonary metastasis are shown in Table 1. Tumors were located in the upper extremities, the lower extremities, and the trunk (including the chest wall, back wall, and buttocks) in 10, 17, and 6 patients, respectively. The most frequent histology was myxofibrosarcoma, undifferentiated pleomorphic sarcoma, rhabdomyosarcoma, and epithelioid sarcoma. Twenty-seven patients (82%) underwent surgery (wide resection and intralesional). Two patients received carbon ion radiotherapy. Four patients received no therapy. There were 19 patients with pulmonary metastasis. The 5-year OS rate of the 33 patients with LNMs was 45.5% (Figure 1).

### 4.2. Association between the Primary Tumor Size and the Timing of LNM Diagnosis

The average tumor size was 75 mm (range 17–183 mm). The average time from the primary tumor diagnosis to LNM was 18.8 months (range 0–144 months). The median time from the primary tumor diagnosis to LNM was 8 months. We investigated if there was a relationship between the primary tumor size and the timing of LNM using Spearman rank analysis; there was no significant correlation found (*Rs* = 0.0088, *p*=0.96).

### 4.3. Association between the Time of LNM Diagnosis and Pulmonary Metastasis

**Figure 2:**
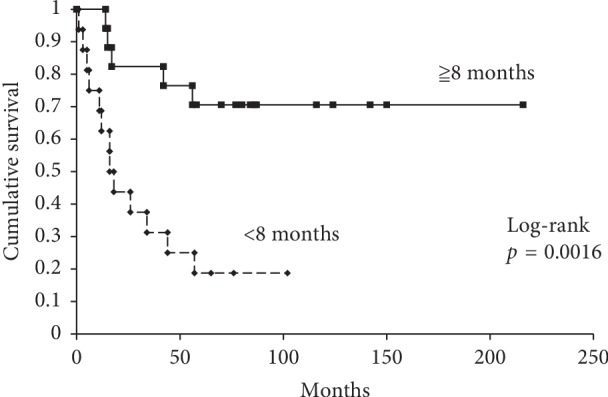
5-year OS rate of patients with time of diagnosis for lymph node metastases of <8 months and ≥8 months.

There were 16 patients with a duration from primary tumor diagnosis to detection of LNM of <8 months (the median time from primary tumor diagnosis to LNM) and 17 with a duration of ≥8 months. Next, we investigated if there was an association between the timing of LNM diagnosis and prognosis. The 5-year OS of patients with a duration of primary tumor diagnosis to LNM of <8 months and those with a duration of ≥8 months was 19% and 71%, respectively (*p*=0.0016) (Figure 2). There were 19 patients with pulmonary metastases. Among them, there were 13 patients with a duration of primary tumor diagnosis to LNM of <8 months and 6 with a duration of ≥8 months (*p*=0.01). The median time from primary tumor diagnosis to pulmonary metastasis was 6 months (range 0–72 months). All LNMs developed earlier than pulmonary metastases, except for 1 patient. Fisher's exact test and Mann–Whitney test were used to compare the timing of the LNM diagnosis and the clinicopathological factors. The following factors were evaluated: sex, tumor size, tumor location (trunk or extremity), presence or absence of surgery, age, tumor grade, and stage. The significant confounding factor was presence or absence of surgery (*p*=0.04). Next, logistic regression was performed for analyzing the relationship between the pulmonary metastases as a dependent variable and the timing of LNM diagnosis as an independent variable. Presence or absence of surgery was used as an independent variable to adjust for confounding factors. The timing of LNM diagnosis was the significant variable (*p*=0.046) (Table 2). The 5-year OS of patients with pulmonary metastasis and those with LNM alone was 17% and 80%, respectively (*p*=0.00019).

### 4.4. Treatment for LNM

**Figure 3:**
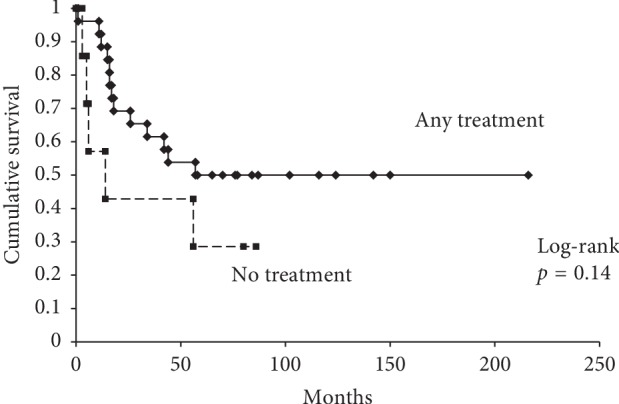
5-year OS rate of patients receiving any treatment and those receiving no treatment for lymph node metastasis.

Surgery was the most common treatment modality for LNM. The number of patients receiving LNM treatment with surgery, surgery + chemotherapy, surgery + radiation, chemotherapy, chemotherapy + radiation, and radiation was 3, 5, 3, 9, 4, and 2. Seven patients received no treatment. Patient demographics are shown in Table 3. The 5-year OS rate of patients receiving any treatment and those receiving no treatment was 50% and 29%, respectively (*p*=0.14) (Figure 3).

## 5. Discussion

The current study revealed that (1) most LNMs occur earlier than pulmonary metastases and (2) survival from LNM depends on the time duration from primary STS diagnosis to detection of LNM, and an early diagnosis of LNM (<8 months) may predict poor prognosis.

The 5-year survival for patients with LNM from STS is reported to be 12.8% to 34.1% [7, 9, 10]. In our study, the 5-year OS was 45.5%. The most common subtype of LNM was myxofibrosarcoma and undifferentiated pleomorphic sarcoma. The low prevalence of lymphogenous histotypes such as rhabdomyosarcoma, epithelioid sarcoma, and clear cell sarcoma may be a result of the small sample size of this study, resulting in relatively good OS. Although myxofibrosarcoma has a high rate of local recurrence, the overall risk of distant metastases is relatively low compared to that of other sarcoma subtypes; the 5-year local recurrence rate has been reported to be 18–31%, with corresponding OS rates of approximately 70% [12–14]. The low risk of distant metastasis is a factor for the relatively good OS associated with myxofibrosarcoma.

Some reports describe that the survival of patients with LNM at diagnosis is similar to that of patients who later develop LNM [10, 11]. Therefore, we examined which time duration from primary diagnosis to detection of LNM influenced OS. Of the 33 patients with LNM, there were 19 patients with pulmonary metastases. All LNMs developed earlier than the pulmonary metastases, except for 1 patient. There were 13 patients with a duration from the primary diagnosis to LNM detection of <8 months and 6 with a duration of ≥8 months (*p*=0.01). The 5-year OS of patients with a duration from the primary diagnosis to LNM detection of <8 months and that of patients with a duration of ≥8 months were 19% and 71%, respectively (*p*=0.0016). Therefore, a short duration from primary diagnosis to LNM detection (<8 months) may predict a shorter survival, as well as possibly representing a component of disseminated disease.

In our study, it was demonstrated that any treatments for LNM did not result in a better survival than that of patients receiving no treatment. The potential benefit of lymphadenectomy remains to be defined. Lymphadenectomy has been shown to improve long-term survival [15, 16], while radical lymphadenectomy does not confer a substantial survival benefit [9, 10]. This study suggests that early LNM (<8 months of duration from the primary tumor diagnosis) may have less impact to decide on an aggressive treatment for LNM.

We acknowledge several limitations of this study: First, there were a limited number of patients with LNM. Second, our study was limited by the fact that imaging assessment (CT or MRI) was used to examine the regional lesions at 3- to 6-month intervals. Therefore, we cannot detect all LNMs unless the patient presents with lymph node swelling. Third, we used a retrospective study design and not a single institutional study design. Fourth, several different histologic tumors were included. Fifth, treatment was not performed in a randomized, controlled fashion.

## 6. Conclusion

This study demonstrated that early LNM (<8 months from the primary diagnosis) may predict poor prognosis.

## Figures and Tables

**Table 1:** Characteristics of the study patients.

	No.
Follow-up period (months)	Median 56 (1–216)

Age at diagnosis (y)	Median 61 (3–89)
<50	7
≥50	26

Gender	
Male	19
Female	14

Tumor site	
Upper extremity	10
Lower extremity	17
Trunk	6

Tumor location	
Superficial	4
Deep	29

Tumor size (cm)	
<5	8
≥5	25

Histology	
Myxofibrosarcoma	8
Undifferentiated pleomorphic sarcoma	6
Rhabdomyosarcoma	5
Epithelioid sarcoma	4
Dedifferentiated liposarcoma	3
Myxoid liposarcoma	2
Pleomorphic liposarcoma	2
Synovial sarcoma	1
Leiomyosarcoma	1
Malignant peripheral nerve sheath tumor	1

Tumor grade	
1	2
2	8
3	23

Surgical stage	
IB	1
IIA	4
IIB	5
III	19
IV	4

Treatment for the primary tumor	
Surgery	27
Carbon ion	2
None	4

Type of surgery	
Intralesional excision	2
Marginal excision	5
Wide excision	20

Pulmonary metastasis	
Absent	14
Present	19

**Table 2:** Results of logistic regression analysis.

	Hazard ratio	95% CI for HR		*p* value
		Lower	Upper	
Surgery: presence	1.114	0.0077	16.116	0.937
LNM: <8 months	6.381	1.031	39.506	0.046

**Table 3:** Characteristics of no-treatment patients for LNM.

	No.
Age at diagnosis (y)	Median 74 (61–79)
<50	0
≥50	7

Gender	
Male	4
Female	3

Tumor site	
Upper extremity	1
Lower extremity	4
Trunk	2

Tumor location	
Superficial	0
Deep	7

Tumor size (cm)	
<5	1
≥5	6

Histology	
Myxofibrosarcoma	2
Undifferentiated pleomorphic sarcoma	1
Epithelioid sarcoma	1
Dedifferentiated liposarcoma	1
Myxoid liposarcoma	1
Malignant peripheral nerve sheath tumor	1

Tumor grade	
1	0
2	4
3	3

Surgical stage	
IB	0
IIA	1
IIB	3
III	2
IV	1

Treatment for the primary tumor	
Surgery	5
Carbon ion	1
None	1

Type of surgery	
Intralesional excision	1
Marginal excision	1
Wide excision	3

Pulmonary metastasis	
Absent	4
Present	3

## Data Availability

The datasets generated and/or analyzed during the current study are available from the corresponding author on reasonable request.
